# Modeling the Interaction of Dodecylphosphocholine Micelles with the Anticoccidial Peptide PW2 Guided by NMR Data

**DOI:** 10.3390/molecules180810056

**Published:** 2013-08-20

**Authors:** Francisco Gomes-Neto, Ana Paula Valente, Fabio C. L. Almeida

**Affiliations:** 1 Laboratory of Toxinology, Instituto Oswaldo Cruz, Fiocruz, Rio de Janeiro 21045-900, Brazil; E-Mail: francisco.neto@ioc.fiocruz.br; 2 Institute of Medical Biochemistry, National Center of Nuclear Magnetic Resonance Jiri Jonas, Federal University of Rio de Janeiro - Institute of Structural Biology and Bioimaging, Rio de Janeiro 21941-920, Brazil; E-Mail: falmeida@cnrmn.bioqmed.ufrj.br

**Keywords:** molecular dynamics simulation, NMR, structure, antimicrobial, membrane, micelles, peptide structure

## Abstract

Antimicrobial peptides are highly dynamic entities that acquire structure upon binding to a membrane interface. To better understand the structure and the mechanism for the molecular recognition of dodecylphosphocholine (DPC) micelles by the anticoccidial peptide PW2, we performed molecular dynamics (MD) simulations guided by NMR experimental data, focusing on strategies to explore the transient nature of micelles, which rearrange on a millisecond to second timescale. We simulated the association of PW2 with a pre-built DPC micelle and with free-DPC molecules that spontaneously forms micelles in the presence of the peptide along the simulation. The simulation with spontaneous micelle formation provided the adequate environment which replicated the experimental data. The unrestrained MD simulations reproduced the NMR structure for the entire 100 ns MD simulation time. Hidden discrete conformational states could be described. Coulomb interactions are important for initial approximation and hydrogen bonds for floating the aromatic region at the interface, being essential for the stabilization of the interaction. Arg9 is strongly attached with phosphate. We observed a helix elongation process stabilized by the intermolecular peptide-micelle association. Full association that mimics the experimental data only happens after complete micelle re-association. Fast micelle dynamics without dissociation of surfactants leads to only superficial binding.

## 1. Introduction

The interaction between proteins and membranes occurs in virtually all biological processes, including the immune response. Antimicrobial peptides constitute the first line of innate immunity and are widespread among the plant and animal kingdoms [1,2]. Structural diversity, cationic nature and amphipathicity are common features of these peptides, allowing them to bind to lipid membranes of diverse chemical natures [2,3,4]. Although the membrane permeation activity of these peptides is well known [5,6], the detailed mechanisms by which they associate with membranes are not fully understood.

PW2 is an anticoccidial peptide (HPLKQYWWRPSI) that has been selected from phage display libraries against living sporozoites of the protozoa *Eimeria acervulina*, the causal agent of coccidiosis, which is an important disease in poultry production [7]. PW2 showed no effects in cell models such as Gram positive and Gram negative bacteria, tumor and kidney cells. However, PW2 was 100% lethal to *Eimeria acervulina* sporozoites. Such high efficiency, in addition to the absence of collateral effects, indicates the potential of PW2 as a model for new anticoccidial drugs.

Paramagnetic relaxation enhancement (PRE) and MD simulation studies on PW2 free in water showed that it is highly flexible, whereas the aromatic region (YWWR) is ordered. T_1_ρ relaxation dispersion experiments on PW2 in water showed that the peptide alternates between conformers at the milli- to microsecond timescale [8], demonstrating the presence of minor stable conformational states in water.

Several studies demonstrate that proteins are in equilibrium between different conformations. While executing a biological function, one conformation is selected by molecular recognition, which is the so-called conformational selection model for binding [9,10,11,12]. A new challenge for structural biology is to describe the energy landscape and discrete conformational states that are biologically relevant.

It is possible to access these conformational states by studying the membrane recognition process and the effect of several different chemical environments on the peptide structure. Our group previously determined the solution structure of PW2 in the presence of sodium dodecyl sulfate (SDS) micelles [13] and dodecylphosphocholine (DPC) micelles [14] and the structural features of PW2 in phosphatidylcholine:phosphatidylethanolamine (PC:PE) vesicles [8]. The aromatic region in the center of the peptide sequence (YWWR) constitutively possesses an identical structure, regardless of the chemical environment. We have shown that membrane recognition by PW2 occurs via conformational selection, where the consensus motif YWWR floats the peptide to the membrane [8,13,14]. However, the N-terminal and C-terminal regions of the peptide display structures that depend on the chemical environment.

In SDS micelles, PW2 adopts a fold in which the aromatic center (YWWR) is protected in a hydrophobic core formed by Trp 7, Leu 3 and Pro 10 [13]. PW2 interacting with PC:PE vesicles showed an ordered aromatic region, which floats the peptide into the lipid vesicle interface [8]. The structure of PW2 in DPC micelles contained an N-terminal 3_10_-helix spanning from Leu 3 to Tyr 6. The aromatic region is well-structured while the C-terminal region (Ser 11 and Ile 12) is flexible [14]. 

For antimicrobial peptides such as PW2, the challenge in structure calculation is the presence conformational exchange that leads to low convergence and geometrical violations in the calculated structures [15,16]. In this case, the calculated structures often do not reflect the biologically relevant conformations.

Here, we performed molecular dynamics (MD) simulations guided by NMR experimental data (NMR structures, dihedral angle and distance restraints) to better understand the events involved in the molecular recognition of an interface. We simulated the association of PW2 with a pre-built DPC micelle and with free-DPC molecules along with spontaneously micelles formation. The simulation with spontaneous micelles formation provided an adequate environment which replicated the experimental data. Hidden discrete conformational states that were averaged in the NMR structure could be described. We observed a helix elongation process stabilized by the intermolecular peptide-micelle association. Full association that mimics the experimental data only happens after complete micelle re-association. Micelle dynamics without dissociation of surfactants leads to only superficial binding. Coulomb interactions are important for initial approximation and hydrogen bonds for floating the aromatic region at the interface, being essential for the stabilization of the interaction. Arg9 is hydrogen bonded with phosphate head group.

## 2. Results

The structure of PW2 in the presence of DPC micelles displays a small N-terminal 3_10_-helix, the conserved aromatic region and a small non-structured C-terminal tail (PDB id 2JQ2) [14]. However, the peptide structure alone is not able to explain its biological activity and the effects on the pathogen membrane, mainly because many of the stabilizing forces are in the intermolecular interaction. Antimicrobial peptides associate with the membrane forming either transmembrane pore that involves interaction with the lipid hydrophobic chains, or superficial association, which forms toroidal pores or induces endocytosis [17,18]. To get insights into the mode of interaction, we performed a series of restrained and unrestrained MD simulations: (i) five different small restrained simulations in the presence of pre-built DPC micelles, to explore the first approach of the peptide to the micelles interface and association/dissociation events; (ii) 100 ns restrained MD simulation in the presence of a collection of free- DPC molecules with spontaneous micelles formation in the presence of the peptide. This simulation gave information on the details of the interaction and also on the longer-term stability of the peptide-interface interaction (iii) 100 ns unrestrained MD simulation in the presence of a collection of free-DPC molecules with spontaneous micelles formation in the presence of the peptide. This simulation gave information on the long-term stability of the peptide structure in the DPC interface environment.

### 2.1. Restrained MD Simulation of PW2 Interacting with a Pre-Built DPC Micelle

**Figure 1:**
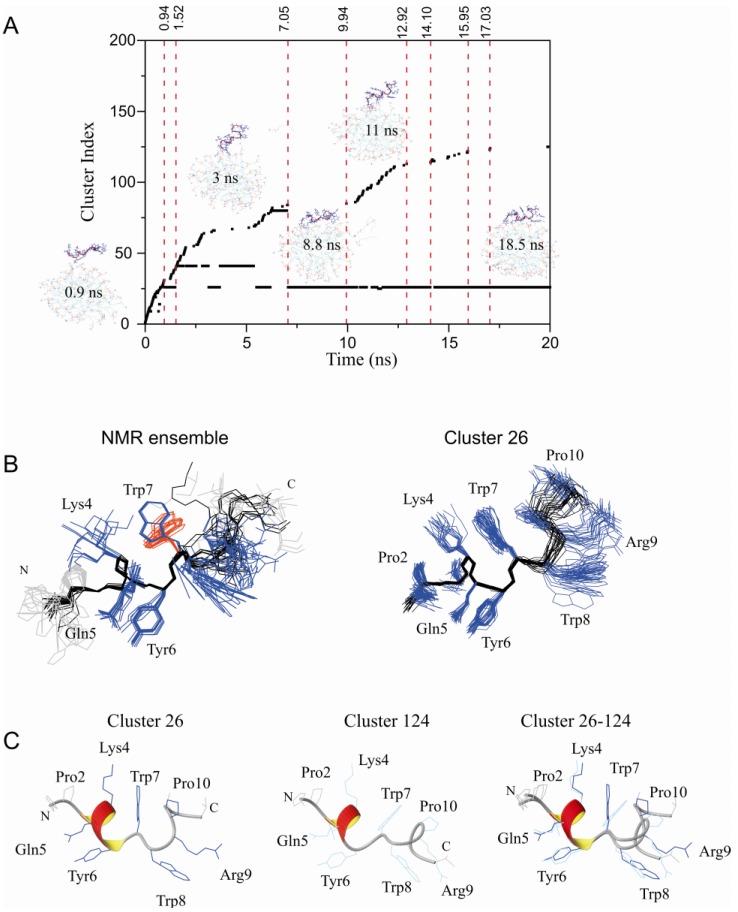
Restrained MD simulation of PW2 interacting with pre-built DPC micelle. (**A**) Cluster index as a function of MD simulation time. Snapshots of interaction are presented at particular simulation times. In the beginning of the MD simulation, there was a rapid increase in the number of detected clusters (0 to 1.5 ns) while the peptide was approaching the membrane surface. The period after this (1.5 and 7 ns) showed binding instability where PW2 was partially bound by the N-terminal His 1 residue. After 7 ns, there was complete interaction of PW2 with the micelle surface, stabilizing cluster 26, and followed by partial dissociation events, indicated by new cluster index increments. Cluster 26 refers to the PW2 structures fully associated with the DPC micelle. (**B**) NMR structure ensemble (PDB ID: 2JQ2), superposition of the 20 lowest energy structures. (right) superposition of 20 representative structures of cluster 26. The backbone is in black and the side chains in blue lines. The second Trp 7 rotamer is in red lines. (**C**) Ribbon representation of clusters 26 (fully bound state, left) and 124 (partially dissociated state, middle) and superposition of both (right), as indicated in the figure. Side chains are represented by dark blue lines for cluster 26 and blue lines for cluster 124.

We ran five different small restrained MD simulations where the peptide was positioned 10 Å from a DPC micelle containing 60 molecules. Cluster analysis of PW2 structures was used to describe the structural details of the PW2-micelle interaction. During the simulation, several conformational states of association were probed. Each new PW2/micelle conformational state generates new cluster index (Figure 1A). Subsequently, we will refer to stabilized structural states by its cluster index.

Upon full association, which occurred in approximately 7.5 ns, cluster 26 was stabilized (Figure 1A,B). During the 20 ns MD simulation run, we probed many partial dissociation events, all of them in the C-terminus of the peptide, and each one generated a new cluster index. Complete re-association of the peptide was frequent and consistently re-formed cluster 26 (Figure 1).

Figure 1B and C shows the comparison between the NMR ensemble (2JQ2) and clusters selected from the MD simulation to represent the peptide in the completely bound conformation (cluster 26) and another ensemble of structures to represent a partially bound conformation (cluster 124). The overall structure is similar with the presence of a 3_10_ helix in the N- terminal. Interesting, while the NMR ensemble shows two rotamers for Trp 7, cluster 26 shows one major rotamer of Trp 7, and the partially bound conformation, represented by cluster 124, displays the other rotamer. Cluster 26 shows one representative discrete bound conformation (Figure 1).

### 2.2. Restrained MD Simulation of PW2 Interacting with free-DPC during the Spontaneous Micellization Showed the Stabilization of a Tighter Micelle-Bound Conformation

**Figure 2:**
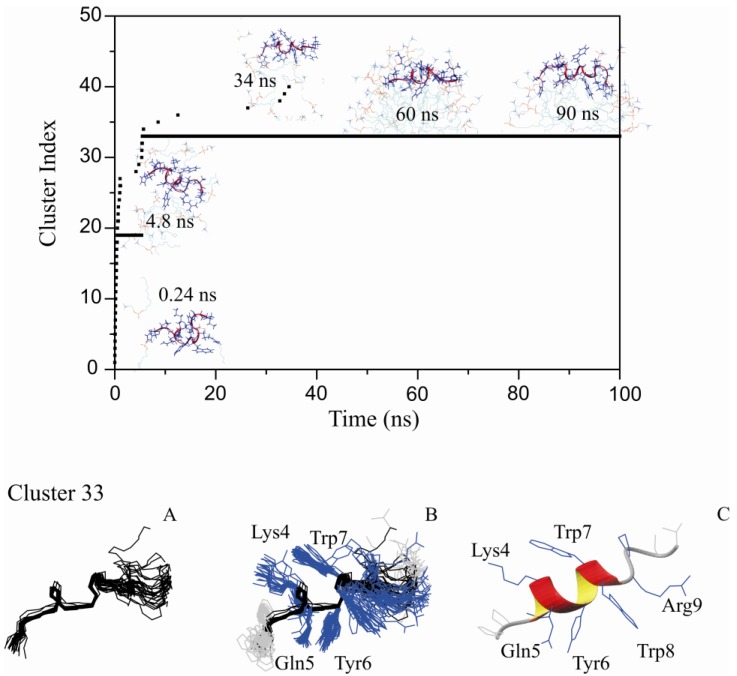
Restrained MD simulation of PW2 interacting with free-DPC during spontaneous micelle formation. (**A**) Cluster index as a function of MD simulation time. Snapshots of interaction are presented at particular simulation times. Initially (0 to 5 ns) there was a fast increase in the number of detected clusters. After 5 ns cluster 33 was stabilized and remained for the entire simulation. Cluster 33 refers to the PW2 structures fully associated with the DPC micelle. (**B**) Superposition of 20 representative structure of cluster 33: backbone (left), heavy atoms (middle) and ribbon representation of the 3_10_-helix (right), spanning from Leu 3 to Trp 8.

Micelles are dynamic supramolecular entities that live for milliseconds. They are constantly exchanging in fast equilibrium between surfactant (DPC) monomers and micelle aggregates [19]. Peptides that associate with micelles are exposed to this complex equilibrium that occurs in the millisecond to second timescale. Peptide molecules are exposed to the micelles but also to the monomeric surfactant. To simulate these millisecond events, we conducted MD simulations of the peptide in a water box containing free-DPC molecules and follow by spontaneous micelle formation and peptide association [20,21]. In these simulations the peptide are always under the influence of the surfactant, at first to the monomers and progressively to micelles. Under this environment, we observed a very stable interaction between PW2 and DPC micelles. Figure 2 shows the PW2 structure cluster analysis for this new chemical environment.

As in the pre-built micelle interaction, in the initial steps of the MD simulation, the cluster index number increased linearly. Upon the formation of a complete micelle aggregate (even before 20 ns), only one stable conformation was detected and identified as cluster 33 (Figure 2, top). Cluster 33 was then observed throughout the MD simulation on, with very few events of partial dissociation. PW2 association during spontaneous micelles formation can be seen as an event for micelles association which account for the micelle equilibrium that occur in milliseconds. Interactions at these conditions were probed for 100 ns and the intramolecular interactions were stable throughout this simulation time. Figure 2 shows an ensemble of 20 structures representing cluster 33, which is similar to the bound conformation represented by cluster 26 (Figure 1). Both cluster 33 and cluster 26 can be seen as discrete bound conformations. We interpreted this results as cluster 26 being an earlier event in the interaction while cluster 26 as a later stage.

### 2.3. Comparison of Clusters 26 and 33

**Figure 3:**
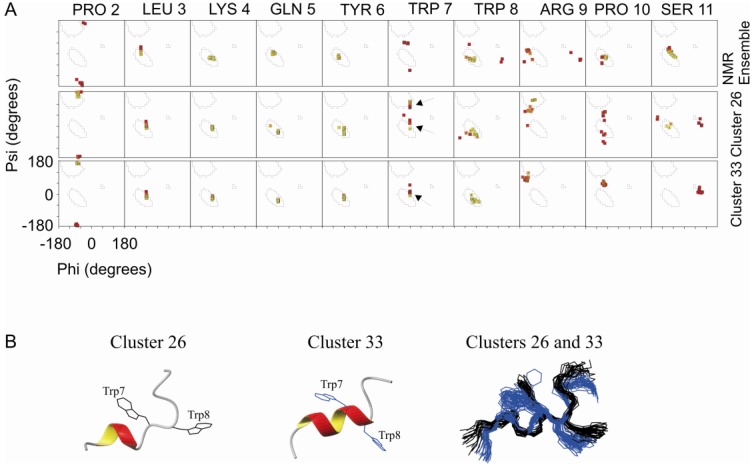
Ramanchadran plot of NMR ensemble, cluster 26 and cluster 33. (**A**) The Ramachandran plot is shown for each amino acid residue of PW2 in the NMR ensemble (top), cluster 26 (middle) and cluster 33 (bottom). The 3_10_-helix spans from Leu 3 to Tyr 6 for NMR ensemble, while in cluster 26 and cluster 33, from Leu 3 to Trp 8. (**B**) Ribbon representation of clusters 26 (left) showing the side chains in black and cluster 33 (middle) in dark blue. In the right, superposition of clusters 26 and 33 showing the same spatial orientation of residues Trp 7 and Trp 8. The arrows indicate the two allowed region of Trp 7 observed for the two clusters.

Figure 3A shows the Ramachandran plots for each residue for the NMR ensemble, cluster 26 and cluster 33. There is a 3_10_-helix that spans from Leu 3 to Tyr 6 in all three conditions. Trp 7 is the breaking point of this helix at the NMR ensemble, whose backbone dihedral angles are in a less favorable region of the Ramachandran plot. For both clusters 26 and 33 we observed a tendency to extend the helix up to Trp8. For cluster 26 the dihedral angles of the Trp 7 backbone are in two allowed regions of the Ramachandran plot, indicated by arrows. In approximately half of the conformations, Trp 7 is in the 3_10_-helix conformation. In these cases, the helix spans from Leu 3 to Trp 8 in the other half from Leu 3 to Tyr 6. For cluster 33, the entire ensemble shows a 3_10_-helix spanning from Leu 3 to Trp 8. Superposition of the ensembles from both cluster 33 and 26 shows very similar structures in both the Trp backbone and rotamer conformation of the side chains with an r.m.s.d. of 0.30 ± 0.09 Å for the backbone of residues 2 to 7 and an r.m.s.d. of 0.99 ± 0.29 Å for the respective side chains (Figure 3B).

We also observed differences between clusters 26 and 33 on the intermolecular interaction, which will be discussed later in the text.

### 2.4. The Unrestrained MD Simulation of PW2 Interacting with Free-DPC Molecules during the Spontaneous Micellization was also Able to Probe the Bound State

We run MD simulations with the NOE potential on the force field was turned off to test the stability of the system without the NMR restraints.

The unrestrained MD simulation in water (data not shown) generated bent structures divergent from the NMR ensemble [8]. PW2 structures are stabilized in a great deal by intermolecular interaction with DPC. Exposure to an environment without DPC “melts” the structures. We observed a similar behavior for the unrestrained MD simulations in the presence of DPC pre-built micelles the peptide was initially immersed in water, 10 Å away from the micelle surface. As a consequence the structure was bent before the complete association.

**Figure 4:**
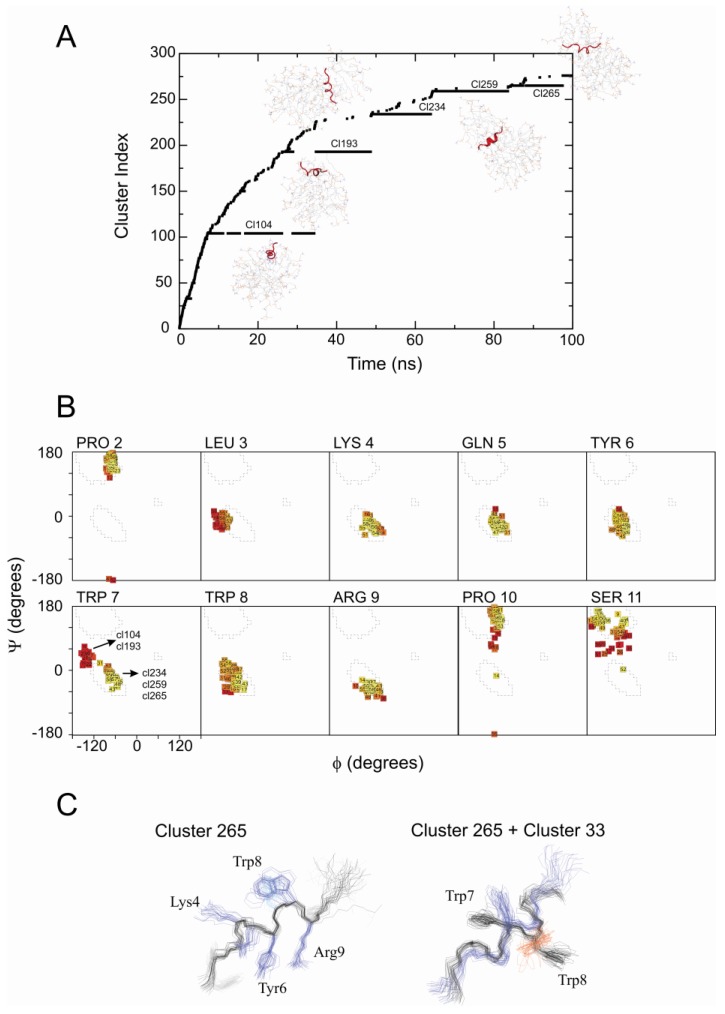
Unrestrained MD simulation of PW2 interacting with free-DPC during spontaneous micelle formation. (**A**) Cluster index as a function of MD simulation time. From 0 to 7 ns we observed an increasing number of clusters. After 7 ns, five long-lived clusters were detected (104, 193, 234, 259 and 265) and representative snapshots of the interaction are shown for each of them. (**B**) Ramachandran plot of all five stable clusters for each amino acid residue. Note that the shift of the Ramanchadran position of Trp 7 from generously allowed region to helix position, indicated by arrows. (**C**) Superposition of 20 representative structures of cluster 265 (left) and ribbon representation of cluster 265 structure (right).

In the absence of NOEs but in the presence of free-DPC molecules, during spontaneous DPC micelle formation, the initial conformation of PW2 peptide was stabilized, maintaining a 3_10_-helix spanning from Leu 3 to Arg 9 up to the end of the MD simulation. The degrees of freedom gained upon suppression of the restraints drove the peptide to a more stable conformation with the stabilization of a longer helix. The unrestrained MD simulation in the presence of free-DPC molecules generated 5 long-lived clusters (104/193/234/259/265, Figure 4A).

In all cluster the peptide structures are very similar. The only differences were observed in the C-terminal residues. The C-terminal residues Pro 10 and Ser 11 display different conformations in their backbone when comparing the NMR ensemble with clusters 26 and 33 (Figure 4B).

The main difference among these bound conformers relies in the way it is interacting with the micelle and not on the PW2 structure. For cluster 104 and 193, PW2 is associated with the interface of a micelle in a similar way that was described for cluster 33, where the main interaction force is a network of hydrogen bonds. For clusters 234, 259 and 265 PW2 is sandwiched between two micelle aggregates. This is a new effect that was not observed for the restrained MD simulation and at this point we do not know whether this is realistic.

### 2.5. Experimental Results Are in Agreement with the MD Simulations

**Figure 5:**
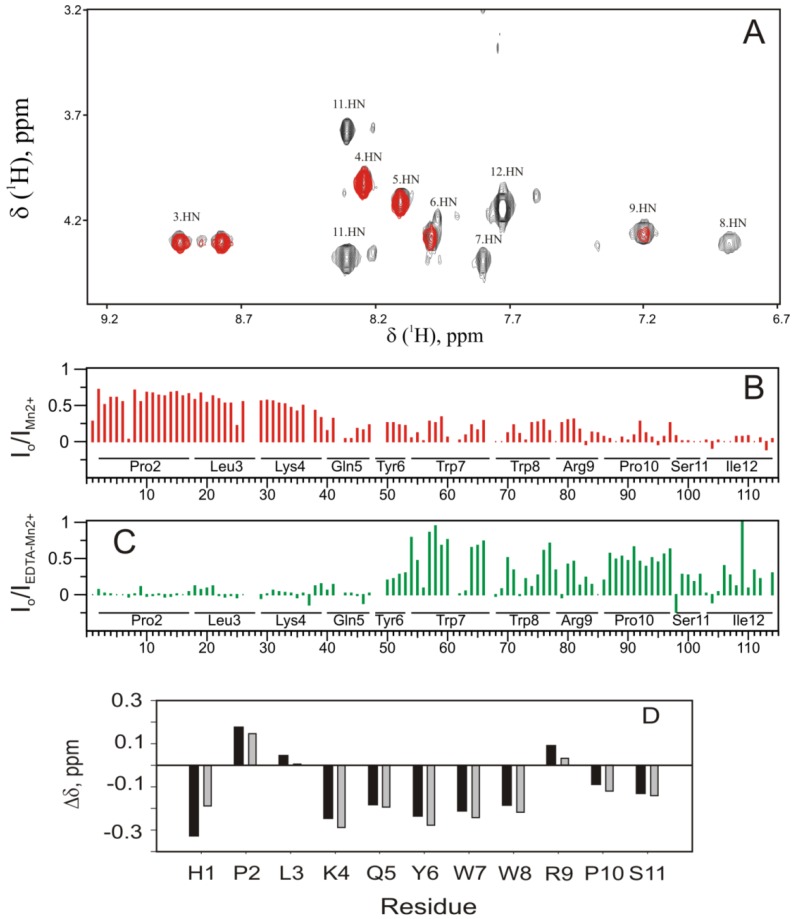
Interaction of the PW2 with DPC micelles. (**A**) Paramagnetic relaxation enhancement (PRE) induced by Mn^2+^ ions. Amide region of a TOCSY spectrum in the absence (black) and presence (gray) of 2 mM Mn^2+^ ions. The resonances of Trp 7, Trp 8, Ser 11 and Ile 12 disappeared and Arg 9 was partially affected, while Tyr 6, Gln 5, Lys 4, Leu 3 were less affected or unaffected. (**B**) Ratio between the TOCSY cross-peaks intensity in presence (I_Mn2+_) and the absence (I_o_) of 2 mM Mn^2+^, expressed as a function of the hydrogen resonance cross-peak assignment (indicated by numeric index and detailed in Table S1). The Mn^2+^ PRE effect was strong for the aromatic region and C-terminal region. (**C**) Ratio between the TOCSY cross-peaks intensity in presence (I_Mn2+/EDTA_) of 2 mM Mn^2+^and 3 mM EDTA and the absence (I_o_), expressed as a function of the hydrogen resonance cross-peak assignment (indicated by numeric index and detailed in Table S1). The Mn^2+^/EDTA PRE effect was strong for the N-terminal region. (**D**) H_α_ chemical shift difference between PW2 in DPC and random coil chemical shifts. Random coil values were according to Wüthrich and coworkers [25,26] and Dyson and coworkers [27,28]. A negative difference suggests the tendency to form a helix from Lys 4 to Trp 8.

To experimentally probe how the PW2-DPC interaction occurs, we measured paramagnetic relaxation enhancement (PRE) induced by the addition of very low concentrations of Mn^2+^ ions into the micelle sample (Figure 5). In the presence of DPC micelles, Mn^2+^ ions associate with the phosphate in the polar head group [22]. When the peptide interacts with the DPC micelle interface, the NMR resonances of each nucleus near the Mn^2+^ ions broadens, showing a decrease in intensity or completely disappearance. In Figure 5A, we show the amide region of a TOCSY spectrum in the absence (black) and presence (gray) of Mn^2+^ ions. Note that the resonances of Trp 7, Trp 8, Ser 11 and Ile 12 disappeared and Arg 9 was partially affected, while Tyr 6, Gln 5, Lys 4, Leu 3 were less affected or unaffected. PRE was quantified by the ratio between the intensity in the presence (I_Mn_^2+^) and absence (I_o_) of Mn^2+^ (Figure 5B and C) and expressed as a function of the hydrogen assignment. In the presence of Mn^2+^, PRE was evident for the aromatic and C-terminal region.

PRE and diffusion coefficients are consistent with the MD simulations. PW2 is always at the micelle surface and floated by the aromatic and C-terminal region. This behavior is simulated by our MD strategy where we probed PW2 association along with spontaneous micelles formation.

We then added EDTA to the mixture to remove the Mn^2+^ ions from the micelle interface and form the soluble complex Mn-EDTA a good paramagnetic probe for water exposed residues [23,24] (Figure 5C). As expected, PRE became more pronounced for the N-terminal residues, which are more exposed to the solvent. The addition of Mn^2+^ did not change PW2 chemical shifts, indicating no direct interaction of Mn^2+^ with PW2.

We also analyzed the helix tendency of PW2 by looking at the hydrogen assignment of PW2 in DPC micelles [14]. Figure 5D shows the difference in chemical shift of PW2 amide resonances with respect to random coil values. Negative difference in the H_α_ chemical shift is indicative of a tendency for helical structure. We observed a helical tendency for residues Lys 4 to Trp 8, Pro 10 and Ser 11. Arg 9 did not show a tendency to form a helical conformation. This result is consistent with the bound conformations represented by cluster 26 and cluster 33, in which a 3_10_ helix span from Leu3 to Trp8.

Additionally, we measured the translational diffusion coefficient (D) by NMR (Table 1). The diffusion coefficient of PW2 free in water is one order of magnitude higher than that of the DPC micelle. Interestingly, the diffusion coefficient of the PW2/DPC system is nearly identical to that of the isolated micelle, suggesting that the peptide is completely bound to the micelle with the association equilibrium shifted toward the bound state. No change in the average micelle size was observed.

**Table 1:** Translational diffusion coefficient for PW2, DPC micelles and the interaction between PW2 and DPC.

	D (m^2^/s)	s.d.
^a^ PW2 _free_	2.63 × 10^−1^°	± 3 × 10^−12^
^b^ DPC_m_	2.62 × 10^−11^	± 4 × 10^−13^
^c^ PW2 + DPC_m_	2.37 × 10^−11^	± 6 × 10^−13^
^d^ PW2 + DPC_m_	2.34 × 10^−11^	± 5 × 10^−13^

^a^ PW2_free_ is the NMR diffusion coefficient of the PW2 peptide free in solution. ^b^ DPC_m_ is the diffusion coefficient of a 300 mM DPC solution (CMC = 1 mM), which is approximately 300-fold higher than the CMC for DPC. ^c^ PW2 + DPC_m_ is the diffusion coefficient of the sample of PW2 interacting with DPC where it was obtained by monitoring the peptide NMR lines. ^d^ PW2 + DPC_m_ is the D coefficient of the sample of PW2 interacting with DPC where it was obtained monitoring the DPC NMR lines.

### 2.6. Analysis and Quantification of the Interaction of PW2 with DPC

**Figure 6:**
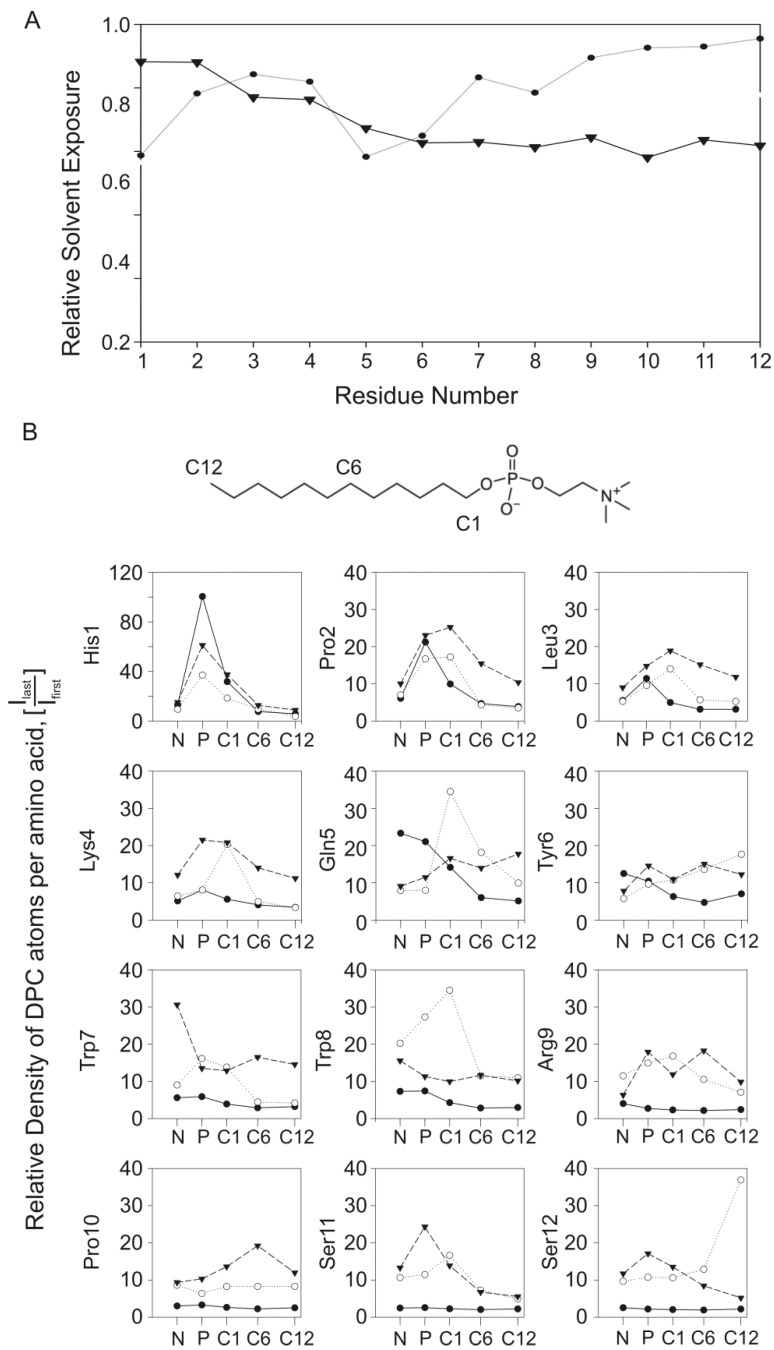
Analysis of PW2 interaction with DPC micelles. (**A**) Relative water exposure of PW2 as function of amino acid residues, for the MD simulations in pre-build DPC micelles (closed circles) and restrained with spontaneous micelle formation (inverted triangle). (**B**) A DPC density profile, g(r), was generated for the center of mass of each amino acid side chain as a function of selected DPC atoms: choline nitrogen (N), polar head phosphate (P), and aliphatic chain atoms C1, C6 and C12. The intensity of the first density peak was plotted as a function of the DPC atom for each MD simulation. The symbols are the same for panel A. The DPC structure illustrates the position of each DPC atom.

To evaluate the forces involved in the interaction, we analyzed the protecting effect of the membrane for the entire peptide structure. The first method consists of measuring the relative water exposure for each residue, by the ratio between the water density in the last (I_last_) and initial nanosecond of simulation (I_first_, Figure 6A). Relative exposures near 1 reflect side chains fully exposed to water, while a decrease reflects restriction in water access.

For the restrained MD simulation of PW2 in the pre-built DPC micelle (Figure 6A, closed circles), we observed high relative water exposure (~0.85) for most residues. The residues His 1 and Gln 5 showed water exposure of approximately 0.6, which indicates that these residues are more protected from the solvent. The 3_10_-helix (Leu 3 to Tyr 6) is touching the micelle surface while His 1 and Gln 5 are deeply associated with the micelle interface. The aromatic region (YWWR) is facing away from the micelle with the C-terminal region almost fully exposed to the solvent and relative water exposure from 0.8 to 1. This result is not in accordance with the observation by PRE, where the N-terminal residues are more exposed to water. We have already hypothesized that these simulations do not reflect the equilibrium situation of the PW2 association with the micelle, but rather the initial events of interaction. That is the reason why His 1 and Gln 5 are the main interactants. They are mainly attracted by coulombic force between the choline phosphate and His 1 and Lys 4.

For the restrained MD simulation of PW2 interacting with free-DPC molecules during spontaneous micellization (Figure 6A, closed inverted triangles), there is a decrease in the relative water exposure for the aromatic and C-terminal region (Gln 5 to Ile 12, relative water exposure below 0.7). The N-terminal residues (His 1 and Pro 2) are exposed while the aromatic region and C-terminal region is in a deeper association with the micelle. The low water exposure of the aromatic and C-terminal region is fully consistent with the PRE experimental results (Figure 5) and could only be probed in the MD simulation with spontaneous micelles formation.

As we claimed earlier, this MD simulation was probing later events in the PW2 association with the micelle and is more compatible with the equilibrium situation probed by the PRE experiment. Milliseconds of MD simulation would be necessary to get information on the PW2 association at equilibrium and the simulation with free-DPC during spontaneous micelles formation worked as a short-cut to obtain information. Analysis of the water exposure for the unrestrained MD simulation of PW2 during spontaneous micellization was not revealing details of interaction because of the formation of PW2 sandwiches. For this reason it is not shown in Figure 6A.

The second method to evaluate the interaction is the density of selected atoms of DPC (N, P, C1, C6 and C12) to the center of mass of each amino acid residue (Figure 6B). For the simulation of PW2 in the pre-built DPC micelle, we observed higher density values for the N-terminal region. His 1 (~100) and Gln 5 (~25) are the actual effectors for the PW2 interaction, which are mainly situated near the phosphate, corroborating the analysis of the relative water exposure. The probability of finding the C-terminus near the interface atoms decreases consistently for all C-terminal residues (from Trp 7 to Ile 12). The low density values for the C-terminal residues reflect the partial dissociation along the simulation. For the interaction probed during spontaneous micellization, His 1 again demonstrates the highest density value (~60), being situated near the phosphate. The hydrophobic amino acids Pro 2 and Leu 3 show peak densities for the C1 DPC atom, which are likely due to the deeper association of His 1. The densities of the DPC atoms near the aromatic and C-terminal region of PW2 increases consistently. These regions are more deeply associated with the micelle and situated close to the aliphatic atoms of DPC.

For the unrestrained MD simulation during spontaneous micellization, the density of the DPC atoms around PW2 is similar when compared with those from the restrained simulation during spontaneous micellization. There is high density at the aromatic and C-terminal region, showing deeper interaction with the micelle interface. There are some differences, such as the high density for Gln 5 and Trp 8. The close proximity of Ile 12 to the C12 atom of DPC is worth noting.

**Figure 7:**
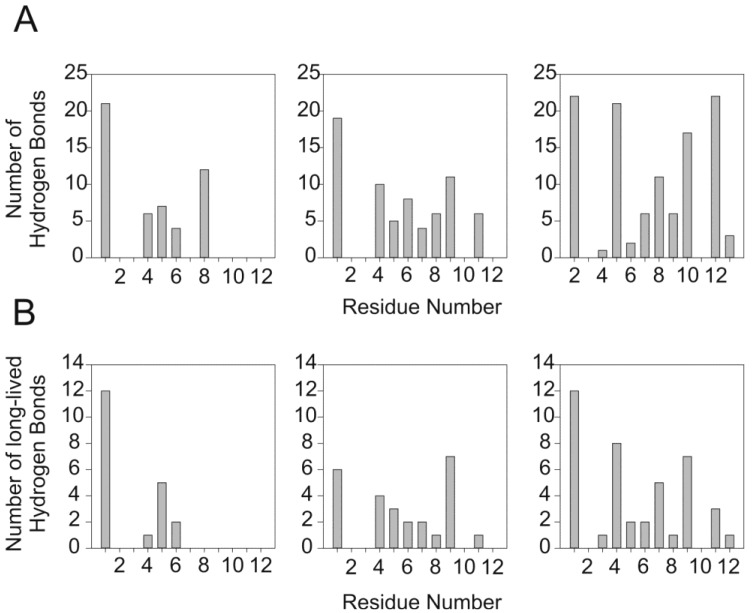
The PW2/DPC interaction depicted by hydrogen bond profile. (**A**) Number of intra-molecular hydrogen bonds (PW2-DPC) for the entire MD simulation as a function of the amino acid residue. The left side is for the simulation in pre-build DPC micelles, the middle for the restrained MD simulation during spontaneous micelle formation and the right for the unrestrained MD simulation during spontaneous micelle formation. (**B**) Number of long-lived intra-molecular hydrogen bonds (PW2-DPC, >1 ns) as a function of the amino acid residue. The left side is for the simulation in pre-build DPC micelles, the middle for the restrained MD simulation during spontaneous micelle formation and the right for the unrestrained MD simulation during spontaneous micelle formation.

The third method to evaluate the interaction is by measuring the hydrogen bonds profile between the peptide and the DPC polar head group (Figure 7). Figure 7A shows the number of hydrogen bonds during the entire time of the MD simulation, and Figure 7C shows the number of long-lived hydrogen bonds (more than 1 ns).

The MD simulation of PW2 interacting with the DPC micelle showed hydrogen bonding for His 1, Lys 4, Gln 5, Tyr 6 and Trp 8. The absence of C-terminal hydrogen bonds is remarkable, which again indicates the partial dissociation observed in the cluster analysis and density profiles. Excluding short-lived hydrogen bonds highlights that His 1 and Gln 5 are the prevalent residues in the initial steps of interaction. Trp 8 hydrogen bonds are short-lived as are many of the hydrogen bonds with Lys 4.

The restrained MD simulation of PW2 during spontaneous micelle formation showed extensive hydrogen bonding from His 1 in the N-terminal region through the entire aromatic and C-terminal regions up to Ser 11 with the exception of Leu 3 and the proline residues (Pro 2 and Pro 10). Excluding short-lived hydrogen bonds maintained the profile, which decreased the number of hydrogen bonds. The major change in the hydrogen bond profile was observed in the number of hydrogen bonds for Arg 9, suggesting an important role for this residue in the interaction.

The unrestrained MD simulation of PW2 interacting with free-DPC molecules showed a similar hydrogen bond profile but with an increased number of hydrogen bonds. In this simulation, His 1 displayed an identical behavior. However, Lys 4, Trp 7 and Arg 8 showed increased numbers of hydrogen bonds due to the nature of the interaction. Here, during the 100 ns of the MD simulation, PW2 is sandwiched between two micelles (clusters 234, 259 and 265, Figure 4A) for approximately 50 ns. Interestingly, Ile 12 showed long-lived hydrogen bonds mainly at the end of the simulation.

## 3. Discussion

To better understand the mechanism for the molecular recognition of DPC micelles by PW2, we performed molecular dynamics simulations guided by NMR data focusing on different strategies to explore the transient character of the interaction. The strategy presented here took into account PW2 association-dissociation equilibrium, and the transient nature of the micelle that is constantly forming and reforming in a millisecond timescale. To achieve this goal, we simulated the binding of the peptide to a pre-built DPC micelles and also to a “soup” of free-DPC molecules where we probed association while the micelles was forming. We have also probed the interaction experimentally using paramagnetic relaxation enhancement and measurement of diffusion coefficient.

From the experimental results we observed a deeper association of the aromatic and C-terminal region of the peptide. The experimental results describe an equilibrium situation, which probably demands seconds to minutes to reach equilibrium. We have previously reported that when a perturbing agent such as other surfactant was added to PW2 in DPC micelles, it takes minutes to re-establish equilibrium [14]. This means that PW2 interaction to DPC does not simply involve a local association with the micelles interface, but also the micelle needs to re-structure in order to reach equilibrium.

We also learned from the diffusion experiments that PW2 is fully associated with the micelles presenting the same diffusion constant as the micelles alone. This is important because it tells us that the flexible conformation of the peptide free in water does not need to be taken into consideration in the MD simulations, since equilibrium is fully shifted toward the associated peptide. In fact on the simulations we observed only partial dissociations. Diffusion constant also informed that the micelles’ size remains the same in the presence of peptide.

From the Hα chemical shifts we learned that PW2 has a tendency to helix in the aromatic region. In fact, structure calculation showed helical structure from residue Leu 3 to Tyr 6, while MD simulation of the fully associated conformation (cluster 33) showed a 3_10_-helix spanning from Leu 3 to Trp 8. The unrestrained MD simulation the helix spanned up to Arg 9. The structural calculation of peptides based on NMR data is guided by the search for an ensemble of structures that individually satisfy the experimental data. Such structures represent individual solutions to a set of constraints rather than necessarily reflecting the ensemble of structures accessible to the peptide in solution. Different conformations may be hidden within an average of states that are sampled in the NMR data.

Even in a situation where multiple NMR restraints are detected, it is difficult to assess the relative populations of these conformations due to the general semi-quantitative character of NOEs [29]. Only tight NOEs contain enough information to assess the relative population of multi-conformer structures, and even in this case, this information must be interpreted with caution. NMR data have only a limited capacity to distinguish between the peptide adopting a single folded conformation as opposed to various mixtures of folded and unfolded conformations [30].

**Figure 8:**
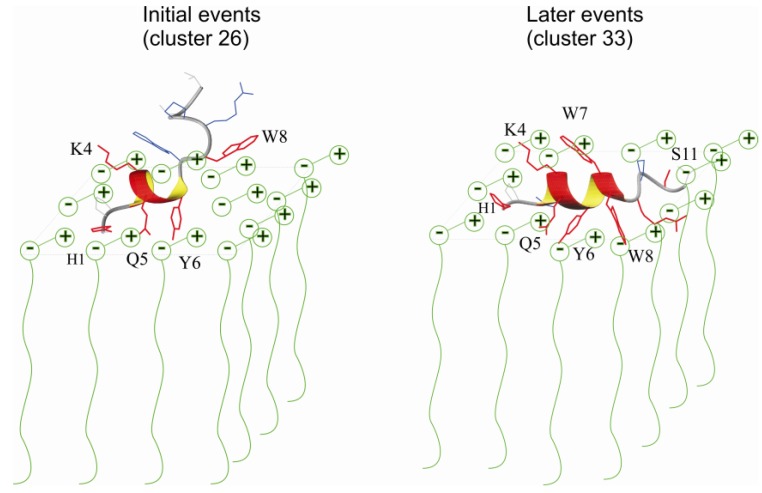
Schematic representation of the proposed model of two events for PW2 association with DPC interface. In the left is a static representation of the association of a cluster 26 structure with the micelle. In red are the residues that are hydrogen bonded with DPC head group during simulation. Note that residues His 1, Lys 4, Gln 5 and Tyr 6 forms long-lived hydrogen bonds, while Trp 8 forms only short-lived hyrogens bonds. Cluster 26 structures interact superficially with the DPC head groups and the C-terminal residues are weakly bound. In the right we represent the association of a cluster 33 structure with DPC micelles. Note that that the helix was elongated if compared with cluster 26. This elongation was only possible in the simulation with free-DPC with spontaneous micelles formation. Our model assumes that this deeper association was only possible after micelle re-equilibration, event that happens in millisecond to second timescale. In red are the residues that are hydrogen bonded with DPC micelles. Note that most of the residues show long-lived hydrogen bonds. Number of intra-molecular hydrogen bonds (PW2-DPC) increased consistently when compared for the entire MD simulation as a function of the amino acid residue.

The MD simulation in the presence of pre-built micelles probed the first touch of the peptide to the micelle interface. These simulations were not biased by the positioning of the peptide. Rather, the peptide was positioned away from the interface. We observed that the first touch of PW2 can be either trough the C- or N-terminal, but few nanoseconds after the first touch is enough to stabilize one discrete conformation, represented by cluster 26 with a short 3_10_-helix spanning from residue 3 to 6. The main peptide-DPC interaction involved Coulomb interaction between the positively charged residues His 1 and Lys4 and DPC phosphate and hydrogen bonds between His 1, Lys 4, Gln 5 and Tyr6 and DPC polar head. In this case the interaction is superficial and the C-terminal residues are weakly associated. Trp 8 is also involved in hydrogen bond with DPC molecules but we observed partial dissociation of the aromatic and C-terminal residues. Figure 8 summarized the observed interactions. In red are the residues interacting by hydrogen bond with the micelles polar head.

The MD simulations in free-DPC with spontaneous micellization resulted in a deeper interaction. PW2 is located at the interface, and there is no dissociation at the C-terminus. The C-terminal residues from Tyr 6 to Ile 12 showed average water exposure of 0.6, which indicates that the entire C-terminal region is more deeply associated with the micelle interface and the aromatic region is fully floated to the micelle. This MD simulation is in agreement with the PRE experimental results (Figure 5), where we observed that the C-terminal region is less exposed to water and deeper in the membrane interface. We related this simulation with latter events of PW2 association that depends on the micelle reorganization.

Similar to the MD simulation that resulted in cluster 33, the unrestrained simulation in free-DPC with spontaneous formation of micelles resulted in an interfacial association of PW2 with the micelle. It is remarkable that the presence of free-DPC provided a chemical environment that stabilized the observed conformation seen for the restrained MD simulation. The association is similar to the observed for cluster 33, with the difference that with the unrestrained conformation we also observed PW2 sandwiched between two micelle aggregates.

Simulation on a pre-built micelle probes early events in the peptide-micelle association. Wennerstrõm *et al*. [31] showed experimentally that nanosecond timescale motions are related mainly to the diffusion of the surfactant monomer within micelle surface. During this fast timescale only small accommodation of the interface related to surfactant diffusion is taking place. Simulations during spontaneous micelle formation probed later events (millisecond events) of peptide-micelle association. Bond *et al*. [21] simulated the properties of the bacterial outer membrane protein OmpA in the presence of DPC. The spontaneous micelles formation was the chosen strategy to simulated micelle formation around OmpA, event that occurs in milliseconds (micelle lifetime [19]) and the strategy enabled the simulation in nanoseconds MD. Similar strategy was used to simulate PW2 association with DPC micelles.

### 3.1. Interaction Forces

We observed PW2 simultaneously interacting with an average of 8–12 DPC molecules. Figure 8 summarizes the two mode of interaction observed. The in-and-out longitudinal motion of the DPC molecules, along its long molecular axis, is very important to accommodate the peptide. This motion, which occurs in pico- to nanoseconds, enables the maximum interaction of the peptide with the DPC interface. The interface is plastic and moldable to the peptide requirements. Even residues that are exposed to the solvent can also interact with the DPC polar head group because the head group can easily enter and exit the micelle surface and form hydrogen bonds with these residues.

The forces that drive the association of PW2 with DPC micelles are mostly long-range Coulomb interactions for the initial events (His 1, Figure S1) and hydrogen bonds with the DPC polar head groups. Hydrogen bonds between His 1 and Gln 5 with DPC are the first stabilizing interaction (Figure S1). Hydrogen bonds that involve residues in the aromatic and C-terminal region are formed at latter steps, as probed by the MD simulation of free-DPC with spontaneous micellization.

The key interaction for stabilizing the deeper association of PW2 with the DPC micelle is the hydrogen bond between Arg 9 and the DPC phosphate group (Figures S2 and S3). Note that this interaction is the most prominent intermolecular long-lived hydrogen bond (>1 ns, Figure 7C) and is not present in the simulation with the pre-built DPC micelle. We also observed other long-lived hydrogen bonds involving residues in the aromatic and C-terminal region, in particular Trp 7, Trp 8 and Ser 11.

### 3.2. Discrete Conformational States of PW2

From all possible conformational states available for PW2, during the MD simulations, we observed the stabilization of two discrete conformational states: cluster 26, which is the ensemble of structures of the superficially bound conformation that reflects the initial events of binding, and cluster 33, which is the ensemble of structures after the equilibrium is reached with the re-accommodation of the micelle and deeper interaction. The structures of both ensembles are similar in that they stabilize the 3_10_-helices, but in latter stages the helix is extended from residues Leu 3 to Trp 8.

Cluster 26 and 33 represents the bound conformations and cluster 33 can be confirmed by experimental evidence of a helix in these regions. Figure 5C shows that the difference between the chemical shift of the alpha hydrogens of PW2 in the DPC micelle [14] and the random coil values is negative, which indicates that a helix spans from Lys 4 to Trp 8.

PW2 structures were only stable in the presence of the DPC interface. The 3_10_-helix was stabilized in all MD simulations in the presence of DPC. These structures are conformational states that were hidden in the averaged NMR ensemble. In the averaged NMR ensemble, Trp 7 was a helix breaking residue likely because of the partial association-dissociation equilibrium that was observed in the MD simulation of PW2 with the DPC micelle, which probed the initial events of the interaction. The association equilibrium was not detected by the NMR ensemble and was probed by restrained MD simulation on the DPC micelle.

Experiments of interaction of PW2 to membrane vesicles shows that PW2 is unable to disrupt (causing leakage) and interact weakly with vesicles formed only with phophatidylcholine (PC). The presence of another phospholipid such as phosphatidylethanolamine (PE) promotes leakage and stronger interaction [8]. Both phospholipids are zwitterionic, but the presence of PE adds dynamics to the polar heads. In the present work we showed that even in micelles, re-accommodation of the system is essential for strong interaction and milliseconds to seconds timescale is needed for interaction.

## 4. Experimental

### 4.1. Chemistry Procedures

#### 4.1.1. Preparation of the Peptide Sample

PW2 (HPLKYWWRPSI) was synthesized commercially by the solid phase method at Genemed Synthesis Inc. (San Antonio, TX, USA). The synthetic peptide was purified by reverse-phase high-performance liquid chromatography (RP-HPLC) and the PW2/DPC NMR sample was prepared to a final concentration of 3 mM PW2, 300 mM DPC-_d38_ (Cambridge Isotope Laboratories, Andover, MA, USA), 20 mM aqueous sodium phosphate buffer (pH 5.0), 100 mM sodium chloride and 10% D_2_O (99.9%, Isotec Inc., Miamisburg, OH, USA).

#### 4.1.2. Peptide Assignment

Peptide assignment is deposited in the Biological Magnetic Resonance Data Bank (BMRB) [14] under the accession number 15,267.

#### 4.1.3. DPC Assignment

The assignment of the DPC resonance lines was performed by analyzing the ^1^H homonuclear bidimensional NMR spectra recorded at 25 °C on a Bruker Avance DRX600 spectrometer operating at 600.04 MHz. TOCSY spectra (spin-lock time of 70 ms) were acquired using the MLEV-17 pulse sequence [32]. NOESY spectra were acquired using a 75 ms mixing time. Water suppression was achieved using the WATERGATE technique [33]. The spectra were collected with 2048 × 512 data points. COSY spectra (1024 × 256 data points) [34] and HMBC spectra (4096 × 160 data points) [35] were acquired.

#### 4.1.4. Analysis of the PW2/DPC Interaction by the Paramagnetic Relaxation Enhancement (PRE) Effect of Mn^2+^ Ions in Solution

The paramagnetic ion manganese (Mn^2+^) interacts with the polar head phosphate of the DPC molecule. The presence of the paramagnetic ion promotes a broadening of the peptide hydrogen resonance lines near the micelle surface, as observed by 2D TOCSY spectra [22], which allows for the identification of the residues that directly interact with the micelle. Adding ethylenediaminetetraacetic acid (EDTA) chelates the paramagnetic Mn^2+^ ions, shifting them from the micelle interface to the bulk water phase such that the line broadening effect is reversed [23].

Manganese binds to negative charges of the system what could be either the negative phosphate of DPC or a negative charge of the peptide.In the experimental condition probed we have used 100× excess of DPC. The only negative charge of PW2 is the carboxy terminal. We than expect and observed a high preference for the manganese ion near the phosphate probing the residues that are near the surface of the micelle.

We performed two experiments: one with the PW2/DPC sample in the presence of 2 mM Mn^2+^ (manganese chloride) at a 2:300 (Mn^2+^:DPC) molar ratio and another that was identical but with an additional 3 mM EDTA (Sigma Aldrich). NMR spectra were recorded at 25 °C on a Bruker Avance DRX600 spectrometer operating at 600.04 MHz. TOCSY spectra (spin-lock time of 70 ms) were acquired using the MLEV-17 pulse sequence [32]. Water suppression was achieved using the WATERGATE technique [33]. The spectra were collected with 4096 data points in F2 and 512 data points in F1. NMR data were processed with the NMRPipe [36]. All NMR spectra were analyzed using NMR view version 4.1.2 [37].

#### 4.1.5. Measurement of the Translational Diffusion Coefficient

Molecules in solution are in rotational and translational diffusion, the specifics of which depend on the physical characteristics of the solvents and solutes, such as shape, size, viscosity and temperature. The DOSY spectra were recorded using the bipolar stimulated echo pulse sequence [38], with 128 accumulations and 8192 data point resolution. We performed two sets of experiments, and for each experiment, we varied the gradient strength as follows: (i) 0.674–26.973 G/cm and (ii) 0.674–16.858 G/cm. The gradient pulse length was 20 ms, and the diffusion time was kept constant (50 ms) with a recycle time of 1.5 s. The diffusion coefficient was calculated by non-linear adjustment of intensity curves as a function of time using the T_1_/T_2_ module of the Topspin 2.1 (Bruker BioSpin) program. The gradient calibration was based on the reference value for H_2_O diffusion in the same sample.

#### 4.1.6. Tendency to Helix-Chemical Shift Difference

The difference in the experimental NMR H_α_ chemical shifts between PW2 in DPC [14] and random coil chemical shifts was calculated using random coil values according to Wüthrich and coworkers [25,26] and Dyson and coworkers [27,28] and plotted as a function of the amino acid residue.

### 4.2. Molecular Dynamics Simulation

#### 4.2.1. General Setup

The solution NMR structure of PW2 interacting with DPC micelles, PDB ID 2JQ2 [14], was used in all the molecular dynamics simulations of the present work. The first structure in the NMR ensemble was selected to perform the MD simulations. The correct side chain and the N- and C-terminal ionization states were calculated by the program PROPKA using pH 5.0 [39].

All molecular dynamics simulations and analysis were performed using the GROMACS v. 3.3 software package [40]. The force field and topologies that were used combined the Berger lipid parameters [41] and the parameters defined in the Optimized Potentials for Liquid Simulations (OPLS) force field [42] as suggested by Peter Tieleman [43]. Water was modeled using the SPC model [44]. All simulations were coupled to an isotropic pressure bath of 1 atm and a heat bath of 298 K using standard coupling schemes. The LINCS [45] and SETTLE [46] algorithms were used to constrain bond lengths. Long range electrostatic interactions were calculated using the particle mesh Ewald (PME) [47] method with a 1.0 nm cutoff for the real space calculation [42]. A cutoff of 1.5 nm was used for the van der Waals interactions. The time step for integration was 2 fs, and the coordinates and velocities were saved every 1 ps. Chloride ions were added to neutralize the system charges (His 1, Lys 4, Arg 9), and 100 mM of sodium chloride was added to provide a better comparison with the experimental data.

The generated models were subjected to an energy minimization procedure using three steps: position restrained steepest descents (harmonic constant of 1,000 kJ·mol^−1^·nm^−2^), steepest descents and conjugate gradient method. After this, using the GROMACS simulated annealing schedule, we performed a gradual increase in temperature, from 5 K to 298 K, under a position restraint potential to maintain the original peptide conformation and micelle setup. The position restraint potential was reduced stepwise from 1000 to 0 kJ·mol^−1^·nm^−2^ in steps of 200 ps. Finally, the system was equilibrated in a 1 ns MD simulation.

#### 4.2.2. NMR Distance and Dihedral Restraints

To perform the restrained MD simulations, the experimental NOE-derived distance restraints and dihedral restraints were used [16,48]. The 315 NOE-derived distance restraints in the CNS format [49] were converted to the GROMACS format. All pseudo-atom notations were excluded, and NOEs involving these atoms were treated under the same index in the GROMACS table. The restrictions were applied to the MD simulations using the NMR structure refinement module of the GROMACS package using a constant potential of 1,000 kJ·mol^−1^·nm^−2^ with conservative force weighting. The 5 dihedral restraints were converted to the GROMACS format, and both distance restraints and dihedral restraints were added as topology in the simulations. The model was set up under identical conditions used in the unrestrained MD simulations described above.

#### 4.2.3. Model Setup of PW2 Interacting with a DPC Micelle

The DPC micelle was pre-built by an MD simulation in which we followed the aggregation of 60 free-DPC molecules in a water box for 50 ns, as described by Tieleman and co-workers [20,50]. The pre-built DPC micelle was then centered in a box with the dimensions 6.4 × 8 × 7.2 nm (368 nm^3^). The peptide was positioned at least 10 Å from the micelle surface and 10,923 water molecules were added (182 water molecules per DPC molecule). Five different models were constructed, with different peptide positions and starting parameters, as the initial random velocity distributions. All models were simulated for 20 ns.

#### 4.2.4. Restrained/Unrestrained MD Simulations of PW2 in the Presence of free-DPC Molecules with Spontaneous Micelle Formation

In these simulations, the initial model was built in a box where 64 free-DPC molecules were randomly added to a simulation box (7 × 7 × 7 nm and 343 nm^3^) with the peptide positioned in the center. Then, 9,203 water molecules were added (~144 water molecules per DPC molecule). For this model, we performed two simulations of 20 ns, and for better statistics, one simulation was extended to 100 ns.

#### 4.2.5. Analysis of MD Simulations

All MD simulations were visualized and images were generated using the VMD-XPLOR 1.6 package [51]. The trajectories were analyzed using the GROMACS package suite programs.

##### 4.2.5.1. Clustering Analysis

We applied the GROMACS “g_cluster” module to calculate the RMS clusters of protein conformations using the method of simple linkage (nearest neighbor) with an r.m.s.d. cutoff of 0.10 nm. The cluster representative structures extracted from the trajectories were analyzed with the MOLMOL 2K.1 program [52]. Structure geometries were analyzed using PROCHECK-NMR [53].

##### 4.2.5.2. Density Analysis

Solvent density was used as an exposure marker in the MD simulations. The water density profile, g(r), was generated using the GROMACS “g_rdf” radial distribution function module for each amino acid side chain. The area below the curve g(r) was integrated for the range of 2 nm for the first (maximum water exposure) and last nanosecond of MD simulations. The ratio between the last and initial nanosecond was used as a measure of the relative water exposure and was plotted as a function of the amino acid side chain.

A DPC density profile, g(r), was generated for the center of mass of each amino acid side chain as a function of the DPC atoms: Choline nitrogen (N4), polar head phosphate (P8), and aliphatic chain C1, C6 and C12. The intensity of the first density peak was plotted as a function of the DPC atom for each simulation system.

## 5. Conclusions

MD simulations guided by NMR experimental data enabled a better understanding of the events involved in the molecular recognition of PW2 to DPC interface. The simulation with spontaneous micelle formation provided the most adequate environment for replicating the experimental data. During association there is a helix elongation process stabilized by the intermolecular peptide-micelle association. The helical form of the peptide is probably the membrane-active conformation.

## Supplementary Materials

Supplementary materials can be accessed at: http://www.mdpi.com/1420-3049/18/6/10056/s1.
