# Supplementary material for: Modeling the Interaction of Dodecylphosphocholine Micelles with the Anticoccidial Peptide PW2 Guided by NMR Data
Source: Molecules. 2013 Aug 20;18(8):10056–80. doi: 10.3390/molecules180810056 (PMC6270265; doi:10.3390/molecules180810056)
Supplement: Supplementary file 1 [file molecules-18-10056-s001.pdf]

Supplementary Materials

Figure S1. Restrained MD simulation of PW2 in the presence of the pre-built DPC micelle. (**A**) Snapshot showing the hydrogen bonds between PW2 and DPC polar heads. Note that several DPC molecules interact with PW2 by hydrogen bond. In this figure we observe hydrogen bond with His 1 and Gln 5, the most prevalent and long-lived in the initial events of interaction. (**B**) PW2 interaction with the DPC micelle. The figure shows only the DPC aliphatic acyl chains and the peptide.

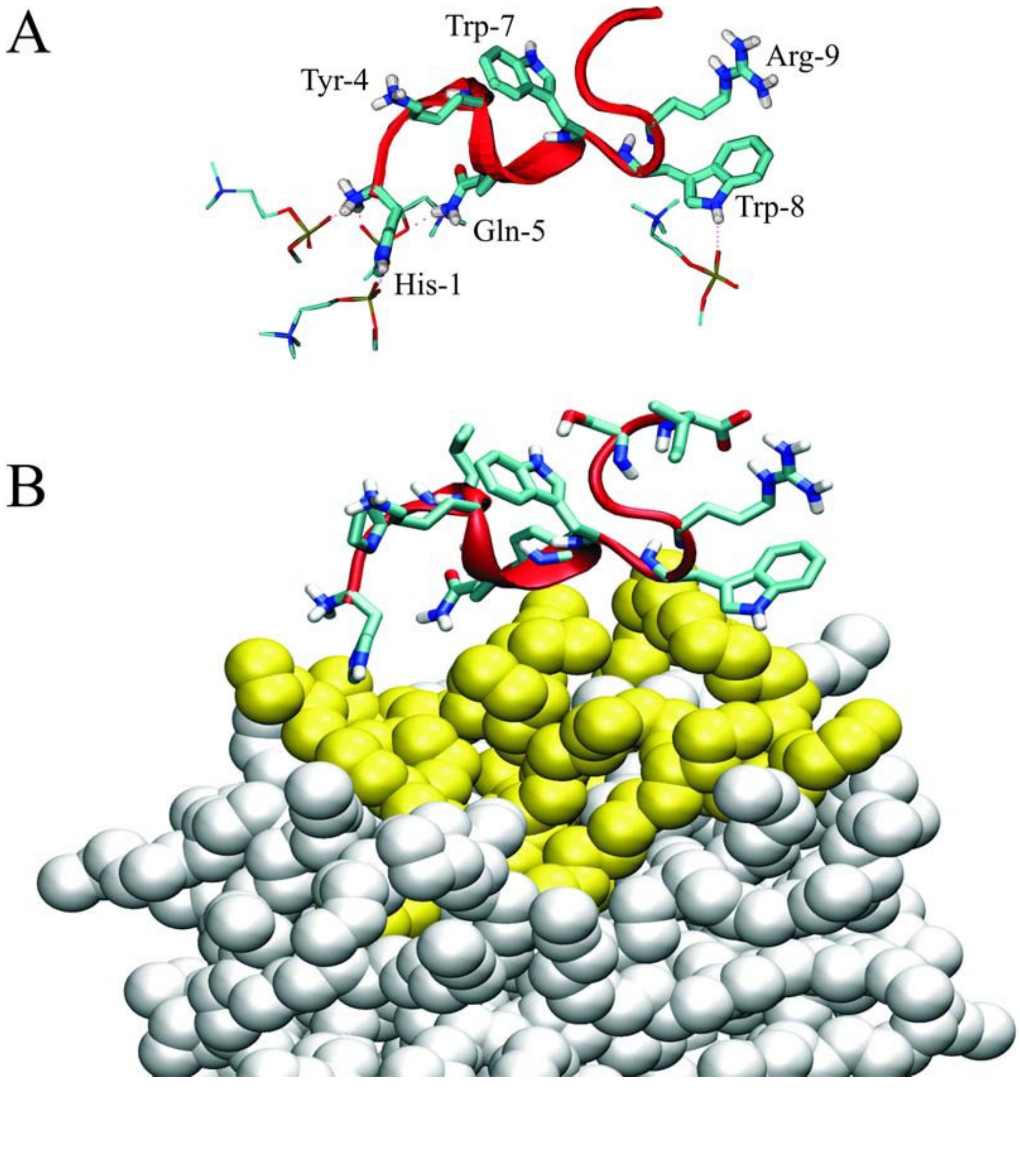

Figure S2. Restrained MD simulation of PW2 in the presence of DPC with free-DPC with spontaneous micelle formation. (**A**) Snapshot of a frame of the MD simulation of PW2 and the hydrogen bonded DPC polar head groups. (**B**) PW2 interaction with the DPC micelle. The figure shows only the DPC aliphatic acyl chains and the peptide. Note that PW2 is more deeply associated with the micelle.

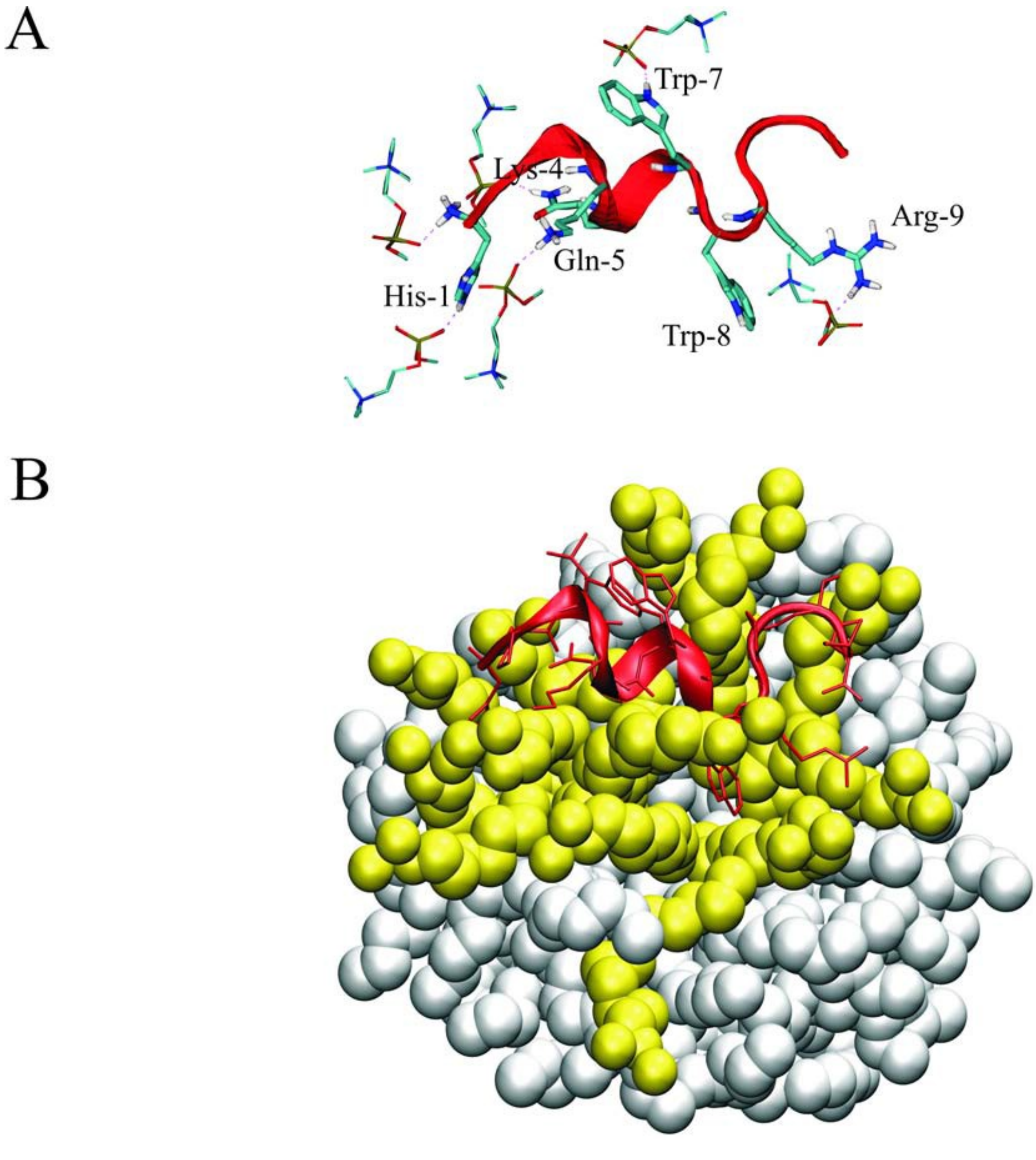

Figure S3. Unrestrained MD simulation of PW2 in the presence of free-DPC with spontaneous micelle formation. (**A**) Snapshot of a frame of the MD simulation of PW2 and the hydrogen bonded DPC polar head groups. Note that PW2 is sandwiched in two micelle interface, forming a network of hydrogen bonds. (**B**) PW2 interaction with the Note that PW2 is more deeply associated with the micelle.

A

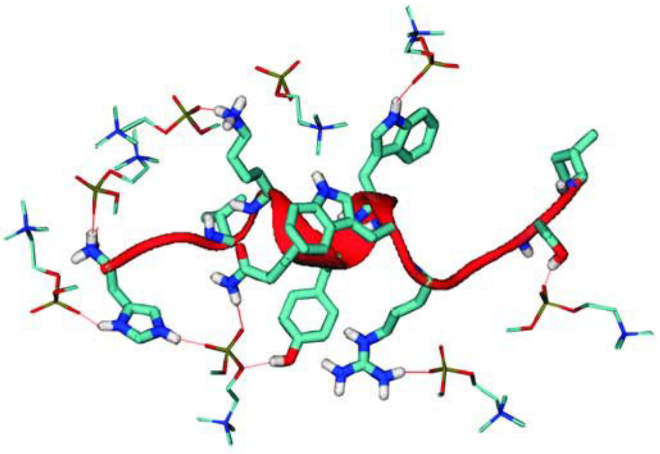

B

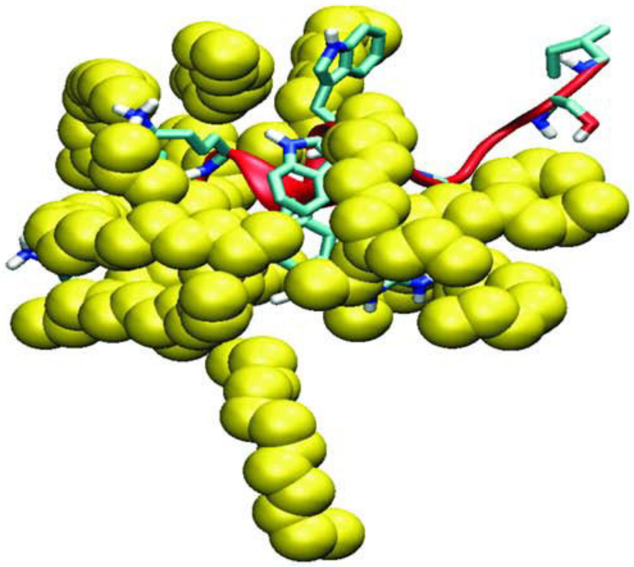

Figure S4. PW2 interaction with the solvent.

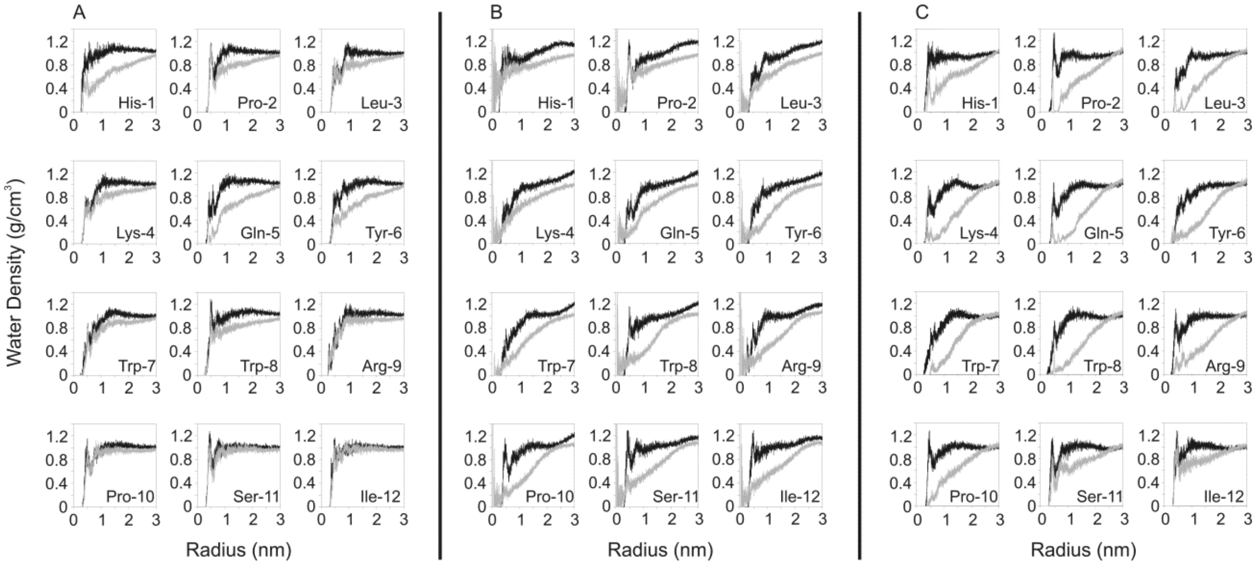

Water density profile, g(r), generated using the GROMACS "g_rdf" module for each amino acid side chain for the 1st nanosecond of the simulation (black) and last nanosecond of the simulation (grey). Note that the water exposure in the last nanosecond is always smaller than in the first nanosecond. To calculate the water exposure for each residues (Figure 7), the area below the curve g(r) was integrated for the range 0-2 nm for the first (black) and last nanosecond of MD simulations (grey). The ratio between the last and initial nanosecond was used as a measure of the relative water exposure and was plotted as a function of the amino acid side chain (Figure 7). "A" refers to the MD simulation in the presence of pre-built DPC micelles, "B" in the presence of free-DPC with spontaneous micelle formation (restrained) and "C" in the presence of free-DPC with spontaneous micelle formation (unrestrained).

Table S1 is linked with Figure 5b, c. The raw data used to build Figure 5 is expressed as function of TOCSY cross-peak assignment. The data was sorted according the residue number and atom names (columns 2 and 3), from N- to C-terminal, receiving a numerical index (column 1, from 1 to 114). The ratio between the TOCSY cross-peaks intensity in absence and the presence of Mn^{2+} is showed in column 4 ($I_0/I_{Mn^{2+}}$). The ratio between the TOCSY crosspeaks intensity in the presence of Mn^{2+}, Mn-EDTA complex is showed in column 5 ($I_0/I_{EDTA\text{-}Mn^{2+}}$).

Table S1. Interaction of the PW2 with DPC micelles.

	**Crosspeak Assignment**		$[I_0/I_{Mn^{2+}}]$	$[I_0/I_{EDTA\text{-}Mn^{2+}}]$
1	1HE1	1HD2	0.28	0
2	2HB	2HG+	0.72	0.07
3	2HB+	2HG+	0.51	0.02
4	2HB1	2HD1	0.61	0.01
5	2HB1	2HD2	0.61	0
6	2HB1	2HA	0.55	0
7	2HD1	2HB1	0.03	−0.03
8	2HD1	2HG1	0.71	0.01
9	2HD1	2HG2	0.55	0.11
10	2HD2	2HD1	0.68	−0.02
11	2HD2	2HG2	0.67	−0.01

Table S1. *Cont.*

	Crosspeak Assignment		$[I_0/I_{Mn^{2+}}]$	$[I_0/I_{EDTA\text{-}Mn^{2+}}]$
12	2HD2	2HD1	0.64	0.01
13	2HD2	2HB1	0.63	−0.03
14	2HD2	2HG1	0.68	−0.02
15	2HG1	2HB1	0.69	0.01
16	2HG1	2HG2	0.63	0
17	2HG2	2HB1	0.66	0.04
18	3HB1	3HD+	0.58	0.12
19	3HB1	3HG	0.67	0.07
20	3HG	3HD+	0.54	0.09
21	3HG	3HB1	0.63	0.12
22	3HN	3HA	0.59	−0.01
23	3HN	3HA	0.53	−0.03
24	3HN	3HA	0.53	−0.01
25	3HN	3HB+	0.22	−0.04
26	3HN	3HB+	0.55	0
27	3HN	3HB+	0.00	
28	3HN	3HB+	0.00	
29	4HA	4HG+	0.56	−0.05
30	4HA	4HB#	0.57	0.01
31	4HB1	4HG1	0.56	0.06
32	4HE1	4HG1	0.53	0.04
33	4HE1	4HD1	0.52	0.03
34	4HN	4HA	0.47	0.02
35	4HN	4HB2	0.42	−0.04
36	4HN	4HG1	0.5	0.02
37	4HZ	4HG1	1.38	−0.14
38	4HZ	4HE2	0.43	0.12
39	4HZ	4HD2	0.33	0.15
40	5HA	5HB+	0.15	0.06
41	5HA	5HG+	0.32	0.14
42				
43	5HE11	5HE21	0.04	0.02
44	5HE21	5HE22	0.04	0.02
45	5HN	5HB1	0.18	−0.01
46	5HN	5HG1	0.16	−0.12
47	5HN	5HA	0.23	0.02
48				
49				
50	6HD1	6HE1	0.26	0.2
51	6HE1	6HD1	0.26	0.22
52	6HN	6HB1	0.23	0.28
53	6HN	6HA	0.22	0.3
54	7H	7H	0.05	0.79
55	7HB1	7HA	0.12	0.47
56	7HE1	7HE1	0.01	0.09
57	7HE3	7HZ3	0.28	0.86
58	7HE3	7HH2	0.26	0.95

Table S1. *Cont.*

	Crosspeak Assignment		$[I_0/I_{Mn^{2+}}]$	$[I_0/I_{EDTA\text{-}Mn^{2+}}]$
59	7HH2	7HZ3	0.34	0.68
60	7HH2	7HZ3	0.06	0.76
61				
62	7HN	7HB+	0.02	0.01
63	7HN	7HA	0.09	0.05
64	7HZ2	7HZ3	0.23	0.65
65	7HZ2	7HH2	0.16	0.68
66	7HZ3	7HH2	0.29	0.74
67				
68	8HB1	8HA	0	−0.02
69	8HE1	8HE1	0	0.08
70	8HH2	8HZ3	0.12	0.51
71	8HH2	8HZ2	0.23	0.34
72	8HN	8HA	0.11	−0.01
73	8HZ2	8HH2	0.02	0.22
74	8HZ2	8HH2	0.26	0.11
75	8HZ2	8HZ3	0.27	0.27
76	8HZ3	8HZ2	0.3	0.61
77	8HZ3	8HH2	0.15	0.71
78	9HE	9HB+	0	0.34
79	9HE	9HG+	0.26	−0.04
80	9HE	9HG	0.3	0.42
81	9HE	9HB+	0.31	0.46
82	9HN	9HE	0.17	0.13
83	9HN	9HB+	−0.04	0.24
84	9HN	9HG+	0.13	0.14
85	9HN	9HA	0.12	0
86	10HB1	10HG1	0.07	0.2
87	10HB1	10HD1	0.04	0.57
88	10HB1	10HA	0	0.49
89	10HD1	10HG1	0.06	0.53
90	10HD1	10HB1	0.02	0.47
91	10HD1	10HD2	0.09	0.66
92	10HD2	2HG2	0.28	0.46
93	10HD2	10HG2	0.12	0.39
94	10HD2	10HB1	0.06	0.51
95	10HD2	10HG2	−0.04	0.45
96	10HD2	10HD1	0.07	0.56
97	10HG1	10HD1	0.26	0.63
98	11HA	11HB#	0.08	-0.24
99	11HB1	11HA	0.01	0.28
100	11HB1	11HA	0.01	0.27
101	11HN	11HB1	0	0.18
102	11HN	11HA	0	0.28
103	12HA	12HD1	0.02	0.02
104	12HA	12HD1	−0.09	−0.11
105	12HA	12HB	0.02	0.04

Table S1. *Cont.*

	**Crosspeak Assignment**		**[$I_0/I_{Mn^{2+}}$]**	**[$I_0/I_{EDTA\text{-}Mn^{2+}}$]**
106	12HB	12HG2	0	0.4
107	12HB	12HG12	0	0.27
108	12HB	12HG11	0.07	0.12
109	12HG11	12HG12	0.07	1.11
110	12HG12	12HG11	0.08	0.09
111	12HN	12HA	0	0.34
112	12HN	12HD+	0.05	0.22
113	12HN	12HG+	−0.11	−0.41
114	12HN	12HB	0.04	0.3
